# Therapiestrategien bei primär kutanen B‐Zell‐Lymphomen – Ergebnisse einer monozentrischen Kohortenstudie an 98 Patienten

**DOI:** 10.1111/ddg.15702_g

**Published:** 2025-07-14

**Authors:** Rohat Cankaya, Pit Leonard Kleiner, Franz Joachim Hilke, Rose Moritz, Thomas Eigentler, Max Schlaak, Gabor Dobos

**Affiliations:** ^1^ Klinik für Dermatologie Venerologie und Allergologie Charité – Universitätsmedizin Berlin; ^2^ Hauttumorzentrum Charité – Universitätsmedizin Berlin

**Keywords:** CBCL, Exzision, kutanes B‐Zell‐Lymphom, kutanes T‐Zell‐Lymphom, Patientenüberleben, prognostische Faktoren, Primär kutanes Lymphom, systemische Therapieoptionen, Strahlentherapie, Time to next treatment (TTNT), Triamcinolon, CBCL, cutaneous B cell lymphoma, cutaneous T cell lymphoma, excision, patient survival, prognostic factors, Primary cutaneous lymphoma, radiotherapy, systemic treatments, time to next treatment (TTNT), triamcinolone

## Abstract

**Hintergrund:**

Primär kutane B‐Zell‐Lymphome (CBCL) sind chronische Erkrankungen mit häufigen Rezidiven. Die Zeit bis zur nächsten Therapie (*Time to next treatment*, TTNT) ist ein Endpunkt, der den klinischen Nutzen von Therapieoptionen einschließlich der Patientenperspektive widerspiegelt. Ziele waren es, klinische Merkmale, Überleben, Prognose und TTNT bei CBCL zu analysieren.

**Patienten und Methodik:**

In dieser monozentrischen Studie wurden klinische Daten von Patienten zwischen 1998 und 2022 erhoben. Die TTNT wurde berechnet. Es wurden univariate und multivariate Analysen durchgeführt.

**Ergebnisse:**

Insgesamt wurden 46 Patienten mit Follikelzentrumslymphom (pcFCL), 41 mit marginalzonen‐lymphoproliferativer Störung (pcMZLPD) und elf mit diffus großzelligem B‐Zell‐Lymphom, Bein‐Typ (DLBCL‐LT) identifiziert. Bei 26% der Patienten mit pcFCL traten mehrere Rezidive auf. Das rezidivfreie 5‐Jahres‐Überleben betrug 71%, 87% beziehungsweise 23% bei pcFCL, pcMZLPD und DLBCL‐LT. Bei pcFCL und pcMZLPD schnitten hautgerichtete Behandlungen wie Exzision oder intraläsionales Triamcinolon am besten ab, während Chemotherapien bei DLBCL‐LT eine mittlere TTNT von 38 Monaten erreichten. In der multivariaten Analyse war eine Beinbeteiligung signifikant mit geringerer TTNT der ersten Behandlung bei allen Patienten assoziiert, während Komorbidität mit höherer TTNT einherging.

**Schlussfolgerungen:**

DLBCL‐LT hatte die schlechteste Überlebensrate. Hautgerichtete Therapieoptionen erreichten tendenziell eine höhere TTNT bei pcFCL und pcMZLPD, während systemische Behandlungen eine höhere TTNT bei DLBCL‐LT aufwiesen.

## EINLEITUNG

Primär kutane Lymphome sind eine heterogene Krankheitsgruppe seltener Non‐Hodgkin‐Lymphome, die entweder von T‐ oder B‐Lymphozyten ausgehen.^1^, ^2^ Die letztgenannte Krankheitsgruppe, die primär kutanen B‐Zell‐Lymphome (CBCL),^1^, ^2^ besteht aus den folgenden Diagnosen: primär kutanes Follikelzentrumslymphom (pcFCL), primär kutane marginalzonen‐lymphoproliferative Störung (pcMZLPD) (auch primär kutanes Marginalzonenlymphom [pcMZL] genannt),^1^ diffus großzelliges B‐Zell‐Lymphom, Bein‐Typ (DLBCL‐LT) und weitere seltene Entitäten.^1^, ^3^, ^4^

Das Konzept des pcMZL wurde in den vergangenen Jahrzehnten mehrfach angepasst. Im Jahr 2005 definierten Willemze et al. das pcMZL als indolentes Lymphom und als Teil der Gruppe der extranodalen B‐Zell‐Marginalzonenlymphome. Das pcMZL besteht aus folgenden Zelltypen: kleinzellige B‐Zellen, marginalzonen‐ beziehungsweise zentrozytenähnliche Zellen, lymphoplasmazytoide Zellen und Plasmazellen.^2^ In der aktuellen Version der WHO‐Klassifikation lymphatischer Neoplasien haben Alaggio et al. das pcMZL aufgrund seiner besonderen klinisch‐pathologischen Merkmale als eigenständige Entität in Abgrenzung zu anderen Marginalzonenlymphomen eingestuft.^3^ Der internationale Konsensausschuss ging sogar noch weiter und stufte das pcMZL aufgrund seines indolenten Verhaltens von einem Lymphom zu einer lymphoproliferativen Störung (pcMZLPD) herab, da die krankheitsspezifische Überlebensrate bei nahezu 100% liegt, ohne dass aggressive Therapien erforderlich sind.^4^ Daher bezeichnen wir die Krankheitsentität als pcMZLPD.^4^

Neben unterschiedlichen Nomenklaturen mangelt es aktuell auch an einem Konsens über die Therapie der pcMZLPD. Neben dem beobachtenden Abwarten (*watchful waiting*) kann eine Behandlung von Patienten mit pcMZLPD aufgrund der möglichen Beeinträchtigung der Lebensqualität durch sichtbare Hautläsionen oder Juckreiz erforderlich sein, was den Behandlungswunsch der Patienten verstärken kann. In dieser Studie wurde nur in Ausnahmefällen *watchful waiting* gewählt, da unseres Wissens Evidenz für ein *watchful waiting* bei pcMZLPD fehlt.^5^, ^6^

Während pcFCL und pcMZLPD als indolente CBCL‐Erkrankungen eine gute Prognose aufweisen, haben Patienten mit DLBCL‐LT nach wie vor eine schlechte Überlebensrate.^7^, ^8^, ^9^, ^10^, ^11^ Kutane B‐Zell‐Lymphome sind chronische Erkrankungen, die sich durch häufige Rezidive im Krankheitsverlauf auszeichnen.^7^, ^8^, ^9^, ^10^, ^11^ Aufgrund der geringen Stichprobengröße klinischer Studien sind die Erkenntnisse über das Gesamtüberleben und das rezidivfreie Überleben (OS, RFS) sowie prognostische Faktoren spärlich.

Nach den aktuellen Empfehlungen stehen zur Behandlung von CBCL hautgerichtete und systemische Therapieoptionen zur Verfügung, die von der Exzision über intraläsionale Kortikosteroide bis hin zur Polychemotherapie reichen.^5^, ^6^

In den letzten Jahrzehnten haben Behandlungsschemata mit Rituximab und Polychemotherapie das Überleben von Patienten mit DLBCL‐LT verbessert.^12^ Im Gegensatz zu primär kutanen T‐Zell‐Lymphomen (CTCL) hat sich die wiederholte Gabe von therapeutischen Wirkstoffen, die bereits bei CBCL‐Patienten eingesetzt wurden, in klinischen Studien als wirksam erwiesen.^13^, ^14^

Da bei Patienten mit CBCL mit Rezidiven während des Krankheitsverlaufs gerechnet werden kann, werden häufig mehrere Therapielinien verabreicht. Aufgrund der wenigen klinischen Studien gibt es nur wenig Evidenz für die Wirksamkeit der Therapieoptionen.

### Zeit bis zur nächsten Therapie

Campbell et al. führten den Endpunkt Zeit bis zur nächsten Therapie (*Time to next treatment*, TTNT) in klinischen Studien über CTCL und chronische Krankheiten ein. Er wird vom Beginn einer Therapie bis zum Beginn einer nachfolgenden Therapielinie gemessen.^15^ TTNT ist ein praktikabler Surrogatmarker, der den klinischen Nutzen, die Patientencompliance und die Toxizität von Therapieoptionen zuverlässig bewertet. Im Jahr 2022 wurde die TTNT in klinischen Studien zu CBCL bislang noch nicht bewertet.

### Prognostische Faktoren

Sowohl klinische als auch immunhistochemische Biomarker wurden in univariaten und multivariaten Analysen als prognostische Faktoren bei CBCL beschrieben, wobei die meisten davon eine Beinbeteiligung^9^, ^16^, ^17^, ^18^ und multiple Läsionen umfassten.^11^, ^16^, ^18^, ^19^, ^20^

Mian et al. berichteten über den *Cutaneous Lymphoma International Prognostic Index* (CLIPI).^19^ In dieser Studie berechneten sie einen Score mithilfe folgender Parameter: erhöhte Laktatdehydrogenase (LDH)‐Werte im Serum, Vorhandensein von Knoten als primäre Hautläsion und eine Gesamtzahl von Läsionen über 2. Bei positiver Beantwortung wurde für jeden Parameter ein Punkt vergeben.^19^

### Zielsetzungen

Ziele dieser Studie waren es, die Patientencharakteristika und das Überleben von CBCL‐Patienten zu bewerten, TTNT zu bestimmen und prognostische Faktoren auf der Grundlage klinischer Parameter zu ermitteln.

## PATIENTEN UND METHODIK

### Aufbau der Studie

In diese monozentrische und explorative Kohortenstudie haben wir Patienten mit CBCL aufgenommen, die älter als 18 Jahre waren und zwischen 1998 und 2022 an der Charité –Universitätsmedizin Berlin behandelt wurden.

Nach der klinisch‐pathologischen Korrelation wurden die Patienten nach den WHO‐EORTC‐Klassifikationen von 2005 und 2018 diagnostiziert.^1^, ^2^

Diese Studie wurde in Übereinstimmung mit den Grundsätzen der aktuellen Fassung der Erklärung von Helsinki durchgeführt und von der institutionellen Ethikkommission genehmigt (Geprüft und genehmigt durch die lokale Ethikkommission Berlin; Genehmigung Nr.: EA1/196/08.)

### Datenerhebung

Die klinischen Daten wurden prospektiv von den behandelnden Ärzten^21^ aus den Krankenakten entnommen und umfassten Patientencharakteristika (Alter, Geschlecht und Begleiterkrankungen), Hautbeteiligung zum Zeitpunkt der Diagnose (Art, Stelle und Ausmaß), T‐Klassifizierung nach Kim et al.^5^ und erhöhte LDH‐Werte.

Weitere Informationen über den Krankheitsverlauf, einschließlich Rückfälle, Krankheitsverlauf und Überleben, wurden gesammelt. Darüber hinaus wurden Daten über den Behandlungsverlauf mit allen verabreichten Therapielinien extrahiert.

Der Leistungsstatus der *Eastern Cooperative Oncology Group* (ECOG) wurde jedem Patienten retrospektiv anhand der Krankenakten zugewiesen.^22^

In Anlehnung an Mian et al. berechneten wir für jeden Patienten einen CLIPI.^19^ Dabei waren zwei Anpassungen erforderlich: Wenn die LDH nicht bestimmt wurde, wurde ein Wert von 0 zugewiesen. Anstatt Patienten mit mehr als zwei Läsionen gemäß Mian et al. zu berücksichtigen, wiesen wir Patienten mit mehr als einer Läsion einen Punkt zu.^19^ Diese Anpassungen wurden als notwendig erachtet, da bei 51 CBCL‐Patienten LDH‐Werte fehlten. Außerdem war es uns nicht möglich, die Anzahl der Läsionen der Patienten zu bewerten, da dieser Parameter in einigen Fällen nicht dokumentiert war.

Die Datenerhebung wurde retrospektiv durchgeführt, während die Follow‐up‐Daten bis zum 30. Juni 2022 gesammelt wurden. Patienten, bei denen die Diagnose nach dem 30. Juni 2022 gestellt wurde, wurden nicht berücksichtigt.

### Zeit bis zur nächsten Therapie

In unserer Studie wurden die standardisierten TTNT‐Definitionen von Campbell et al. beachtet.^15^ Zur Berechnung der TTNT in unserer Kohorte wurden folgende Anpassungen als notwendig erachtet: Zwei identische Therapielinien wurden als zwei getrennte Linien betrachtet, wenn die Unterbrechung zwischen den identischen Therapielinien länger als 12 Monate dauerte. Wenn der Beginn einer Therapielinie nicht bekannt ist, wurde das Datum des Absetzens der vorherigen Therapielinie zur Bestimmung der TTNT verwendet.

### Statistische Analyse

Statistische Analysen wurden in Microsoft Excel für Mac (Version 16.74) und in IBM SPSS Statistics (Version 29) durchgeführt.

Die Gesamtüberlebenszeit (OS) wurde als Zeit zwischen dem Datum der Diagnose und dem Datum des Todes oder der letzten Nachuntersuchung definiert. DLBCL‐LT‐Patienten galten als verstorben, wenn dies dokumentiert war oder der letzte Besuch in unserem Zentrum mehr als fünf Jahre zurücklag.

Die Unterschiede zwischen den Untergruppen wurden anhand einer univariaten Analyse mit der Methode von Kaplan und Meier und dem Log‐Rank‐Test bewertet. Folgende Parameter wurden bewertet: Geschlecht, Alter (≥ 70 Jahre; <70 Jahre), klinisch‐pathologische Diagnose, CLIPI‐Score, erhöhte LDH‐Werte, Tumorknoten, Ort der Hautläsionen und T‐Klassifikation bei Diagnose. Endpunkte waren OS und rezidivfreies Überleben (RFS).

Die multiple Regressionsanalyse wurde mit den abhängigen Variablen TTNT der Erstlinientherapie und Anzahl der Rezidive gemäß der statistischen Empfehlung von Field durchgeführt.^23^ Variablen, die Unterschiede mit einem p‐Wert < 0,2 aufwiesen, wurden in die multivariate Analyse aufgenommen, wobei fehlende Daten listenweise gelöscht wurden. Weitere Variablen (siehe Datenerhebung) wurden systematisch aufgenommen. Nach der Identifizierung eines signifikanten Modells wurde eine schrittweise Entfernung der Variablen durchgeführt, um die Robustheit des Modells zu bestätigen. Schließlich wurden Multikollinearität und Heteroskedastizität geprüft.

Alle (zweiseitigen) Tests wurden als statistisch signifikant angesehen, wenn der p‐Wert < 0,05 betrug.

## ERGEBNISSE

### Patientencharakteristika

Insgesamt wurden 98 CBCL‐Patienten identifiziert. Ihre klinischen Merkmale sind in Tabelle 1 dargestellt.

**TABELLE 1:** Klinische Merkmale von 98 Patienten mit primär kutanen B‐Zell‐Lymphomen.

Charakteristika	Patienten							
	*Alle Patienten*		*pcFCL*		*pcMZLPD*		*DLBCL‐LT*	
	*Absolut/total*	*%*	*Absolut/total*	*%*	*Absolut/total*	*%*	*Absolut/total*	*%*
**Gesamtzahl**	98		46		41		11	
**Geschlecht, Verhältnis Männer zu Frauen**	54/44		27/19		21/20		6/5	
**Alter, Mittelwert (SD) [Bereich], Jahre**	56 (16) [20–88]		53 (12) [21–77]		48 (17) [20–80]		76 (7) [65–88]	
**ECOG‐Leistungsstatus**								
0	57/61	93	33/33	100	20/22	91	4/6	67
1	1/61	2	0/33	0	1/22	5	0/6	0
2	1/61	2	0/33	0	0/22	0	1/6	17
3	1/61	2	0/33	0	1/22	5	0/6	0
4	0/61	0	0/33	0	0/22	0	0/6	0
5	1/61	2	0/33	0	0/22	0	1/6	17
**Komorbidität**	74/98	76	36/46	78	30/41	73	8/11	73
Immunologisch	28/98	29	13/46	28	15/41	37	0/11	0
Infektiös	12/98	12	5/46	11	5/41	12	2/11	18
Zweitmalignome	18/98	18	10/46	22	6/41	15	2/11	18
Andere	63/98	64	30/46	65	25/41	61	8/11	73
**Ausmaß der Hautbeteiligung bei der Erstdiagnose**								
Eine Körperregion	69/98	70	39/46	85	22/41	54	8/11	73
Zwei Körperregionen	9/98	9	3/46	7	5/41	12	1/11	9
Mehr als zwei Körperregionen	13/98	13	3/46	7	9/41	22	1/11	9
**T‐Klassifikation bei Erstdiagnose** ^a^								
1	36/98	37	19/46	41	12/41	29	5/11	45
2	29/98	30	15/46	33	10/41	24	4/11	36
3	16/98	16	3/46	7	12/41	29	1/11	9
**Art der Hautläsionen**								
Makula	3/98	3	1/46	2	2/41	5	0/11	0
Papel	4/98	4	2/46	4	2/41	5	0/11	0
Plaque	16/98	16	8/46	17	6/41	15	2/11	18
Knoten	53/98	54	21/46	46	24/41	59	8/11	73
**Erhöhter LDH‐Wert bei Erstdiagnose**	14/51	27	5/28	18	1/16	6	3/11	27
**CLIPI**								
0	31/98	32	18/46	39	11/41	27	2/11	18
1	45/98	46	20/46	43	21/41	51	4/11	36
2	21/98	21	8/46	17	9/41	22	4/11	36
3	1/98	1	0/46	0	0/41	0	1/11	9
**Erstlinientherapie**								
Exzision	34/83	41	14/40	35	14/34	41	6/9	67
Strahlentherapie	14/83	17	11/40	28	3/34	9	0/9	0
Triamcinolon (intraläsional)	7/83	8	4/40	10	3/34	9	0/9	0
Topische Steroide	12/83	14	6/40	15	6/34	18	0/9	0
Interferon‐alpha	5/83	6	2/40	5	3/34	9	0/9	0
R‐CHOP	3/83	4	0/40	0	1/34	3	2/9	22
CHOP	1/83	1	0/40	0	0/34	0	1/9	11
Rituximab	4/83	5	3/40	8	1/34	3	0/9	0
**Rezidivrate**	43/98	44	20/46	43	18/41	44	5/11	45
**Mehrfachrezidivrate** ^b^	21/98	21	12/46	26	7/41	17	2/11	18
**Rezidivfreies 5‐Jahres‐Überleben,%**	72		71		87		23^c^	
**5‐Jahres‐Gesamtüberleben,%**	92		100		100		55	

*Abk*.: pcFCL, primär kutanes Follikelzentrumslymphom; pcMZLPD, Primär kutane marginalzonen‐lymphoproliferative Störung; DLBCL‐LT, diffus großzelliges B‐Zell‐Lymphom, Bein‐Typ; SD, Standardabweichung; ECOG, *Eastern Cooperative Oncology Group*; LDH, Laktatdehydrogenase; CLIPI, *Cutaneous Lymphoma International Prognostic Index*; R‐CHOP, Rituximab, Cyclophosphamid, Doxorubicinhydrochlorid, Vincristinsulfat, Prednison;

CHOP, siehe vorherige Abkürzung.

^a^ T‐Klassifikation der Patienten nach Kim et al. TNM classification system for primary cutaneous lymphomas other than mycosis fungoides and Sezary syndrome: a proposal of the International Society for Cutaneous Lymphomas (ISCL) and the Cutaneous Lymphoma Task Force of the European Organization of Research and Treatment of Cancer (EORTC). *Blood*. 2007.

^b^ Patienten mit mehr einem Rezidiv.

^c^ Hier: 5‐Jahre progressionsfreies Überleben.

In jeder Krankheitsgruppe wurden mehr Männer als Frauen identifiziert. Das Durchschnittsalter bei der Diagnose betrug 53 Jahre bei pcFCL, 48 Jahre bei pcMZLPD und 76 Jahre bei DLBCL‐LT. Begleitkrankheiten (Komorbidität) und sekundäre Malignome wurden bei 74 von 98 und bei 18 von 98 CBCL‐Patienten festgestellt.

Der ECOG‐Score konnte retrospektiv bei 61 Patienten ermittelt werden, von denen 57 einen Score von 0 aufwiesen. Bei den Patienten mit pcMZLPD hatten zwei Patienten Scores von 1 und 3, während zwei DLBCL‐LT‐Patienten Scores von 2 und 5 aufwiesen.

Bei 39 von 46, 22 von 41 und 8 von 11 Patienten mit pcFCL, pcMZLPD und DLBCL‐LT zeigte sich eine Hautbeteiligung in einer einzigen Körperregion.

Zum Zeitpunkt der Diagnose befanden sich 19 von 46 pcFCL‐Patienten, 12 von 41 pcMZLPD‐Patienten und 5 von 11 DLBCL‐LT‐Patienten im T1‐Stadium.

Tumorknoten als Erstmanifestation wurden bei 21 von 46, 24 von 41 und 8 von 11 Patienten mit pcFCL, pcMZLPD beziehungsweise DLBCL‐LT festgestellt. 4 von 11 DLBCL‐LT‐Patienten wiesen eine Tumorulzeration auf.

Eine Erhöhung der LDH wurde bei 5 von 28 Patienten mit pcFCL und 1 von 16 Patienten mit pcMZLPD festgestellt. LDH‐Werte wurden dabei nur bei 28 pcFCL‐ und 16 pcMZLPD‐Patienten bestimmt. Darüber hinaus wurde die LDH bei 3 Patienten mit DLBCL‐LT ermittelt, die alle erhöhte Werte aufwiesen.

Die Rezidivraten betrugen 20 von 46 pcFCL‐Patienten, 18 von 41 pcMZLPD‐Patienten und 5 von DLBCL‐LT‐Patienten, wobei Mehrfachrezidive bei 12 von 46, 7 von 41 und 2 von 11 Patienten in jeder entsprechenden Untergruppe auftraten.

Die mittlere Follow‐up‐Zeit betrug 69 (0–282) Monate (Standardabweichung: 66).

Bei einem Patienten mit DLBCL‐LT wurde 31 Monate nach der Diagnose eine pulmonale Manifestation festgestellt. Daher wurde der Patient als M1 eingestuft.^5^ Der Patient verstarb 6 Monate nach Fortschreiten der Erkrankung. Bei einem anderen Patienten mit DLBCL‐LT wurde 11 Monate nach der Diagnose eine nodale Manifestation in den axillären Lymphknoten festgestellt (N1‐Klassifizierung^5^). Der Überlebensstatus dieses Patienten ist unbekannt.

### Überleben der Patienten

Das 5‐Jahres‐RFS betrug 71%, 87% beziehungsweise 23% bei pcFCL, pcMZLPD und DLBCL‐LT, während das 5‐Jahres‐OS bei pcFCL und pcMZLPD 100% und bei DLBCL‐LT 55% betrug.

### Zeit bis zur nächsten Therapie

Tabelle 2 zeigt die TTNT der Therapieoptionen, die die Patienten erhielten.

**TABELLE 2:** Zeit bis zur nächsten Therapie von Therapieoptionen beim primär kutanen B‐Zell‐Lymphom.

Therapieoptionen bei CBCL			Durchschnittliche TTNT (SD) [Monate]		
	*n [Patienten]*	*n [Therapielinien]*	*pcFCL*	*pcMZLPD*	*DLBCL‐LT*
Exzision	39	45	37 (37)	54 (45)	30 (15)
Strahlentherapie	30	34	30 (43)	66 (86)	13 (10)
Triamcinolon (intraläsional)	20	22	29 (25)	24 (46)	–
Topische Steroide	31	34	19 (16)	20 (25)	–
Interferon‐α	14	16	15 (20)	18 (11)	
R‐CHOP	5	5	–	–	12 (13)
CHOP	2	2	–	–	38 (52)
Rituximab	14	16	50 (40)	7 (2)	23 (37)

*Abk*.: n [Patienten], Anzahl der Patienten, die die entsprechende Therapieoption mindestens einmal erhielten; n [Behandlungslinien], Anzahl der Therapielinien der jeweiligen Therapieoption; CBCL, primär kutanes B‐Zell‐Lymphom; pcFCL, primär kutanes Follikelzentrumslymphom; pcMZLPD, primär kutane marginalzonen‐lymphoproliferative Störung; DLBCL‐LT, Diffus großzelliges B‐Zell‐Lymphom, Bein‐Typ; R‐CHOP, Rituximab, Cyclophosphamid, Doxorubicinhydrochlorid, Vincristinsulfat, Prednison; CHOP, siehe vorherige Abkürzung; TTNT, Zeit bis zur nächsten Therapie; SD, Standardabweichung; –, nicht angewandt oder nicht möglich zu bestimmen, da die Stichprobe nur eine Person umfasste.

Folgenden Therapieoptionen wurden als hautgerichtete Therapien (SDT) betrachtet: Exzision, Strahlentherapie, intraläsionales Triamcinolon (TRIL) und topische Steroide, während Interferon (IFN)‐α, Rituximab und Chemotherapie (Cyclophosphamid, Doxorubicinhydrochlorid, Vincristinsulfat, Prednison [CHOP] und CHOP mit Rituximab [R‐CHOP]) systemische Therapien darstellten. Die Dosis der Strahlentherapie variierte zwischen 20 und 36 Gy.

Bei pcFCL waren die Therapieoptionen mit den höchsten durchschnittlichen TTNT Rituximab (50 Monate, Bereich: 9–548), Exzision (37 Monate, Bereich: 4–526) und Strahlentherapie (30 Monate, Bereich: 3–588). Intraläsionales Triamcinolon hatte eine durchschnittliche TTNT von 29 (8–308) Monaten. In der Untergruppe der pcMZLPD erzielten Strahlentherapie, Exzision und TRIL mit 66 (4–880), 54 (0,4–461) beziehungsweise 24 (4–649) Monaten die höchsten durchschnittlichen TTNT. Bei DLBCL‐LT‐Patienten erreichten CHOP, Exzision und Rituximab die längsten durchschnittlichen TTNT mit 38 (4–326), 30 (8–211) beziehungsweise 23 (1–286) Monaten (Tabelle 2).

### Univariate Analyse

**ABBILDUNG 1:**
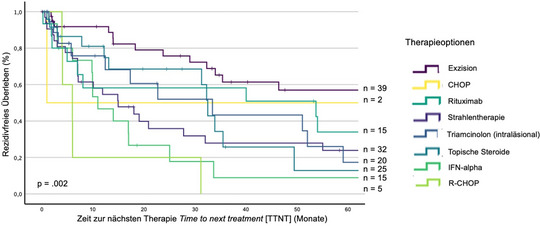
Kaplan‐Meier‐Kurve über das rezidivfreie Überleben von Behandlungsoptionen bei primärem kutanem B‐Zell‐Lymphom (univariate Analyse). Das rezidivfreie Überleben aller Behandlungslinien, die bei Patienten mit primärem kutanem B‐Zell‐Lymphom angewendet wurden, wurde analysiert. *Abk*.: CHOP, Cyclophosphamid, Doxorubicinhydrochlorid, Vincristinsulfat, Prednison. IFN‐alpha, Interferon‐alpha. R‐CHOP, Rituximab, Cyclophosphamid, Doxorubicin‐Hydrochlorid, Vincristinsulfat, Prednison. p, p‐Wert (Signifikanzniveau: α = 0,05). n, Anzahl der Behandlungslinien.

In der univariaten Analyse nach der Kaplan‐Meier‐Methode wurde die 5‐Jahres‐RFS berechnet, die für jede verabreichte Therapieoption wie folgt aussah: Exzision (58%), CHOP (50%), Rituximab (34%), Strahlentherapie (24%), TRIL (18%), topische Steroide (15%), IFN‐α (10%) und R‐CHOP (0%) (p*  =  *0,002) (Abbildung 1).

**ABBILDUNG 2:**
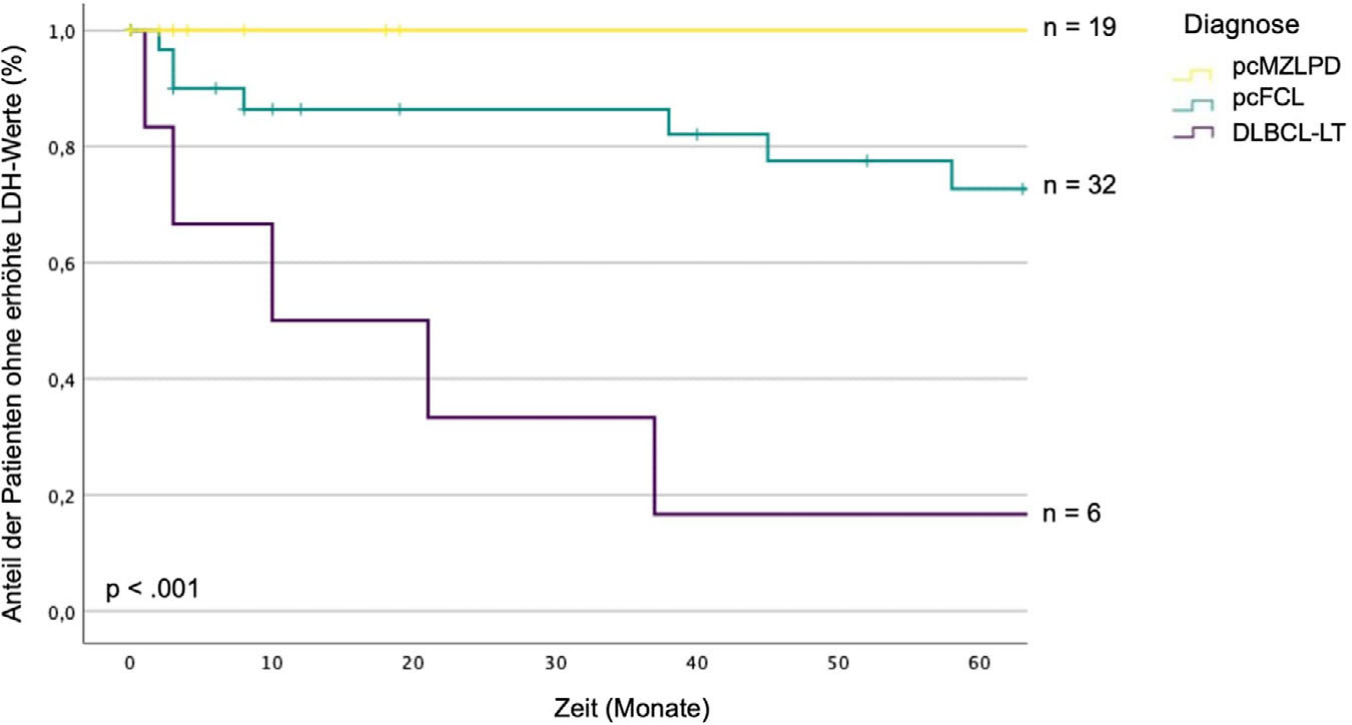
Kaplan‐Meier‐Kurve über erhöhte Laktatdehydrogenase‐Spiegel bei primärem kutanem B‐Zell‐Lymphom (univariate Analyse). Die Unterschiede bei erhöhten Laktatdehydrogenase‐Werten werden zwischen den klinisch‐pathologischen Diagnosen des primären kutanen B‐Zell‐Lymphoms veranschaulicht. *Abk*.: LDH, Laktatdehydrogenase. MZLPD, Marginalzonen‐Lymphoproliferationsstörung. FCL, Follikelzentrum‐Lymphom. DLBCL, LT, diffuses großzelliges B‐Zell‐Lymphom, Beintyp. p, p‐Wert (Signifikanzniveau: α = 0,05). n, Anzahl der Patienten.

In einer weiteren univariaten Analyse hatten 26% der Patienten mit pcFCL und 83% der Patienten mit DLBCL‐LT erhöhte LDH‐Werte. Bei allen Patienten mit pcMZLPD lagen die LDH‐Werte im Normbereich (p < 0,001) (Abbildung 2).

Weitere univariate Analysen zeigten signifikante Unterschiede in der Arm‐ und Beinbeteiligung zwischen den klinisch‐pathologischen Diagnosen innerhalb der CBCL (Abbildungen S1, S2 im Online‐Supplement).

Zwischen den Untergruppen, die nach Geschlecht, Alter, erhöhten LDH‐Werten, CLIPI‐Score, Tumorknoten, T‐Klassifikation und Lokalisation der Läsionen stratifiziert wurden, wurden keine signifikanten Unterschiede bei OS und RFS festgestellt.

### Prognostische Faktoren

In der multivariaten Analyse hatten die Beinbeteiligung aller Patienten bei Erstdiagnose, pcFCL als Diagnose und Komorbidität einen statistisch signifikanten Einfluss auf die TTNT der Erstlinientherapie.

Topische Steroide als Erstlinientherapie, Rumpf‐ und Unterkörperbeteiligung (Körperregionen unterhalb des Nabels, definiert von Kim et al.^5^) bei Erstdiagnose und sekundäre Malignome waren statistisch signifikant für die abhängige Variable Anzahl der Rückfälle (Tabelle 3).

**TABELLE 3:** Multivariate Analysen der Zeit bis zur nächsten Therapie und der Anzahl der Rezidive beim primär kutanen B‐Zell‐Lymphom.

Parameter	Multivariate Analyse			
	*R* 2	*Koeffizient*	*B*	*p*
**Zeit bis zur nächsten Therapie, erste Therapielinie**				
Beteiligung der Beine bei Erstdiagnose	19,2%	−125,737	−0,287	0,013
Primär kutanes Follikelzentrumslymphom		−90,885	−0,284	0,013
Komorbidität, immunologisch		78,593	0,220	0,043
Komorbidität, nicht infektiös oder immunologisch		96,351	0,280	0,010
**Anzahl der Rezidive**				
Topische Steroide als Erstlinientherapie	35,0%	−2,362	−0,406	0,005
Beteiligung des Unterkörpers bei Erstdiagnose		−1,818	−0,451	0,004
Sekundäre Malignome		−1,277	−0,317	0,022
Rezidivfreie Überlebenszeit		0,009	0,178	0,184
Beteiligung des Rumpfes bei Erstdiagnose		1,064	0,301	0,036

*Abk*.: R^2^, korrigiertes R‐Quadrat; Koeffizient, Regressionskoeffizient B; B, standardisierter Koeffizient B; p, p‐Wert (Signifikanzniveau: α  =  0,05).

## DISKUSSION

Anhand dieser Kohorte von CBCL‐Patienten berichten wir über aktuelle Daten zu Patientenmerkmalen, prognostischen Faktoren und Ergebnissen verschiedener Therapieoptionen unter Anwendung des klinischen Endpunkts TTNT.

Unsere Patientencharakteristika sind vergleichbar mit Kohorten, die zuvor in der Literatur beschrieben wurden, insbesondere im Bezug auf Alter, Geschlecht und Ausmaß der Hautbeteiligung.^8^, ^11^, ^24^, ^25^ Wir identifizierten jedoch in allen Untergruppen mehr Männer, was mit den Ergebnissen von Zinzani et al.^11^ und Lucioni et al.^24^ übereinstimmt, aber den Ergebnissen von Hamilton et al.^8^ widerspricht.

Lucioni et al. wiesen nach, dass Knoten und Plaques die beiden häufigsten Hautläsionen bei pcFCL und DLBCL‐LT sind, was mit unseren Ergebnissen übereinstimmt.^24^

Wir stellten signifikante Unterschiede bei den erhöhten LDH‐Werten zwischen den Krankheitsgruppen fest (Abbildung 2), die auch von Grange et al. gefunden wurden.^16^ Unsere Studie zeigte, dass erhöhte LDH‐Werte bei pcFCL häufiger als bei pcMZLPD sind, worüber bislang in keiner Studie berichtet wurde.

Hallermann et al. berichteten, dass drei von 21 (14%) DLBCL‐LT‐Patienten Tumorulzerationen aufwiesen, während bei pcFCL‐Patienten keine Ulzerationen gefunden wurden. In unserer Kohorte wiesen 36% der Patienten mit DLBCL‐LT Ulzerationen auf.^26^

Andere Kohorten zeigten vergleichbare Überlebensraten zwischen 85%–98%,^9^, ^10^, ^11^, ^16^, ^24^, ^25^, ^26^, ^27^ 90%–100%^9^, ^10^, ^11^, ^25^, ^27^ und 14%–73%^7^, ^8^, ^9^, ^10^, ^11^, ^18^, ^20^, ^24^, ^25^, ^26^, ^27^, ^28^ bei pcFCL, pcMZLPD und DLBCL‐LT. Frühere Studien zeigten ähnliche Rezidivraten zwischen 14%–47%^7^, ^8^, ^9^, ^10^, ^11^, ^16^, ^24^, ^25^ für pcFCL, 36%–57%^7^, ^8^, ^9^, ^10^, ^11^, ^25^ für pcMZLPD und 16%–85% ^7^, ^8^, ^9^, ^10^, ^11^, ^18^, ^20^, ^24^, ^25^ für DLBCL‐LT.

Auf der Grundlage der TTNT scheinen SDT bei pcFCL und pcMZLPD wirksamer zu sein, während systemische Therapieoptionen bei DLBCL‐LT vorteilhafter zu sein scheinen. Eine Ausnahme ist Rituximab bei pcFCL mit einer TTNT von 50 Monaten. Allerdings wurde Rituximab nur sieben Patienten verabreicht. Eine Subanalyse ergab, dass zwei von drei pcFCL‐Patienten mit T3‐Klassifikation Rituximab erhielten, während fünf von 28 pcFCL‐Patienten mit T1‐ oder T2‐Klassifikation Rituximab erhielten. Dies ist bemerkenswert, da pcFCL‐Patienten mit ausgedehnter Hautbeteiligung für Rituximab ausgewählt wurden. Systemisches Rituximab wurde auch als wirksame und gut verträgliche Therapieoption für indolente CBCL‐Entitäten vorgeschlagen,^13^, ^14^ was mit der Wirksamkeit von Rituximab bei unseren pcFCL‐Patienten mit ausgedehnter Hautbeteiligung übereinstimmt. Bei unseren pcMZLPD‐Patienten betrug die mittlere TTNT von Rituximab jedoch nur 7 Monate.

In ähnlicher Weise wurden bei Mogamulizumab, einem monoklonalen Antikörper mit antikörperabhängiger zellulärer Zytotoxizität, höhere Ansprechraten bei CTCL‐Patienten mit höherer Tumorlast beobachtet.^29^ In der univariaten Analyse stellten wir fest, dass die geringste Rezidivrate bei Patienten zu finden waren, die die Therapieoptionen Exzision und Rituximab erhielten. Aufgrund des retrospektiven Studiendesigns können wir jedoch keine definitiven Rückschlüsse auf ihre Wirksamkeit ziehen. Außerdem hängt die Wahl der Therapie von patientenzentrierten Merkmalen, ärztlichen Entscheidungen, Krankenhausstandards und unterschiedlichen nationalen Leitlinien ab.

In der multivariaten Analyse erwies sich Komorbidität als günstiger prognostischer Faktor. Patienten mit Komorbidität zeigen möglicherweise eine höhere Therapiecompliance, was zu längerer TTNT führen könnte. Rice et al. untersuchten den Einfluss der Komorbidität auf die TTNT und das Überleben beim multiplen Myelom. Sie berichteten, dass Patienten mit Asthma oder chronisch obstruktiver Lungenerkrankung (COPD) eine signifikant längere durchschnittliche TTNT der Erstlinientherapie aufwiesen als Patienten, die weder Asthma noch COPD hatten.^30^ In der multivariaten Analyse sind Beinbeteiligung und pcFCL ungünstige Faktoren für die TTNT der Erstlinienbehandlung. In diese Analyse wurden alle CBCL‐Patienten einbezogen, die eine Beinbeteiligung aufwiesen, unabhängig von ihrer Krankheitsentität. Da DLBCL‐LT‐Patienten in der Regel eine Beinbeteiligung aufweisen,^7^, ^8^, ^9^, ^10^, ^11^ könnte dieses Ergebnis die Aggressivität der Erkrankung unterstreichen. Allerdings wurden auch Patienten mit pcFCL und pcMZLPD in diese Analyse einbezogen, was ebenfalls auf eine negative Auswirkung der Beinbeteiligung bei diesen Patienten hinweisen könnte.

In früheren Studien wurde die Beteiligung der Beine ebenfalls als negativer Prognosefaktor identifiziert.^9^, ^16^, ^17^, ^18^ Wir fanden heraus, dass topische Steroide als Erstlinientherapie, die Beteiligung der unteren Körperhälfte bei Erstdiagnose und sekundäre Malignome günstige Prognosefaktoren sind, die die Anzahl der Rezidive beeinflussen. Der Befund zu topischen Steroiden steht in gewissem Widerspruch zu den Ergebnissen aus Tabelle 2 und Abbildung 1, die die TTNT und RFS von topischen Steroiden darstellen. In dieser multivariaten Analyse wurde jedoch ein anderer Endpunkt als TTNT und RFS bewertet. Unsere Erkenntnis, dass die Beteiligung des Unterkörpers ein günstiger Faktor ist, steht in gewissem Widerspruch zu Studien, in denen die Beinbeteiligung in multivariaten Analysen bezüglich des Überlebens der Patienten als ungünstiger Faktor identifiziert wurde.^9^, ^16^, ^17^, ^18^ Die Rumpfbeteiligung ist in unserer Kohorte ein ungünstiger prognostischer Faktor, was mit den Ergebnissen von Smith et al. übereinstimmt.^17^ In unserer Analyse haben wir jedoch den Endpunkt Anzahl der Rückfälle bewertet.

In unserer Kohorte zeigte der CLIPI keine signifikanten Unterschiede im RFS, im Gegensatz zu Mian et al.^19^ Die Autoren fanden heraus, dass der CLIPI einen signifikanten Einfluss auf das progressionsfreie 5‐Jahres‐Überleben bei den indolenten CBCL‐Entitäten hat und eine Risikostratifizierung zwischen pcFCL‐ und pcMZLPD‐Patienten ermöglicht.^19^ Fehlende LDH‐Werte bei 51 Patienten schränkten diese Analyse jedoch ein, da die Patienten hinsichtlich ihres CLIPI aufgrund der von uns vorgenommenen Anpassungen unterschätzt worden sein könnten. Patienten mit potenziell erhöhten LDH, deren Werte jedoch nicht laborchemisch bewertet wurden, könnten einen niedrigeren CLIPI erhalten haben, was zu einer Verzerrung des RFS führt.

Individuelle Therapieentscheidungen, auch wenn sie in multidisziplinären Tumorboards getroffen werden, und das retrospektive Studiendesign können die direkte Vergleichbarkeit zwischen den Therapien einschränken. Wir glauben dennoch, dass die Größe der Kohorte und die Einbeziehung aller Therapielinien die Ergebnisse in Anbetracht der Seltenheit der Krankheit unterstreichen können.^21^ Alle Patienten wurden nach der klinisch‐pathologischen Korrelation als derzeitiger Goldstandard diagnostiziert. Unseres Wissens wurde die TTNT noch nicht in Kohortenstudien bei CBCL zusammen mit Triamcinolon untersucht. In einer Fallserie wurde über intraläsionales Triamcinolon bei CBCL berichtet, die die praktische Anwendbarkeit von Triamcinolon unter anderem betonte.^31^ In dieser Fallserie wurden intraläsionale Kortikosteroide bei neun CBCL‐Patienten untersucht, von denen vier eine vollständige Remission erreichten. Bei den übrigen fünf Patienten wurden partielle Remissionen festgestellt.^31^

### Schlussfolgerungen

In dieser Studie gewannen wir neue epidemiologische Erkenntnisse über CBCL, bewerteten das Überleben der Patienten und ermittelten potenzielle prognostische Faktoren. Diese Studie ist die erste, die Therapieoptionen und ihre Wirksamkeit in einer großen Kohorte von CBCL‐Patienten anhand des Surrogatmarkers TTNT untersuchte. Die Bestimmung dieses kürzlich definierten Endpunkts kann eine nützliche Methode sein, um Therapieoptionen aus praktischer Sicht im klinischen Setting zu bewerten.

Im Vergleich zu systemischen Therapien erwies sich die hautgerichtete Behandlung bei pcFCL und pcMZLPD auf der Grundlage der TTNT als überlegen, während systemische Behandlungen eine höhere TTNT bei DLBCL‐LT erzielten. Eine Ausnahme ist Rituximab, das bei Patienten mit pcFCL und hoher Tumorlast die längste TTNT aufwies.

Derzeit ist intraläsionales Triamcinolon nicht in den deutschen S2k‐Leitlinien für kutane Lymphome aufgeführt.^32^ Triamcinolon ist eine etablierte Therapieoption in der Dermatologie, mit der Patienten mit hypertrophen Narben, Keloiden, diskoidalem Lupus oder vernarbter Alopezie häufig behandelt werden. Aufgrund der Bekanntheit und Erfahrung mit Triamcinolon bei Dermatologen scheint es auch bei CBCL‐Patienten eine wirksame und gut verträgliche Behandlung zu sein. Daher liefert diese Studie Evidenz für intraläsionale Triamcinolon‐Injektionen, was sie zu einer praktikablen Therapieoption macht, insbesondere für CBCL‐Patienten mit multiplen Läsionen.

Auf der Grundlage dieser Ergebnisse unterstützen wir die Nachsorgeempfehlungen der aktuellen deutschen S2k‐Leitlinie für CBCL‐Patienten, um mögliche Veränderungen der Hautbeteiligung zu beurteilen.^32^ Bei pcFCL‐ und pcMZLPD‐Patienten mit weniger als zehn Läsionen empfehlen wir hautgerichtete Therapien wie Strahlentherapie, Exzision oder intraläsionales Triamcinolon. Bei pcFCL‐ und pcMZLPD‐Patienten mit besonders hoher Tumorlast, das heißt mit zehn oder mehr Läsionen, Ulzerationen oder Patienten, die gegenüber früherer Therapielinien refraktär sind, schlagen wir Rituximab oder eine Chemotherapie vor, zum Beispiel CHOP, möglicherweise in Kombination mit Rituximab. Für Patienten mit stark ulzerierten Knoten oder DLBCL‐LT‐Patienten empfehlen wir CHOP oder bei älteren Patienten eine Chemotherapie aus Cyclophosphamid, Vincristinsulfat und Prednison (CVP), die beide mit Rituximab kombiniert werden können. Individuelle Entscheidungen bezüglich der Strahlentherapie für DLBCL‐LT‐Patienten können ebenfalls in Betracht gezogen werden.

Aufgrund des retrospektiven Studiendesigns und gelegentlich fehlender Daten können wir keine eindeutigen Schlussfolgerungen über die Wirksamkeit der Therapieoptionen ziehen. Die vorgestellten Ergebnisse könnten jedoch die klinische Entscheidungsfindung unterstützen.

Multizentrische prospektive Studien sind notwendig, um die Einschränkungen monozentrischer Studien zu überwinden und allgemein gültige (Therapie‐)Empfehlungen zu ermöglichen, wie das *PROCLIPI‐Register* für CTCL^33^ oder das *ADOReg‐Register* für Hautkrebs.^34^

## DANKSAGUNG

Open access Veröffentlichung ermöglicht und organisiert durch Projekt DEAL.

## INTERESSENKONFLIKT

Keiner.

## Supporting information



Supplementary information

## References

[1] Willemze R , Cerroni L , Kempf W , et al. The 2018 update of the WHO‐EORTC classification for primary cutaneous lymphomas. Blood. 2019;133(16):1703‐1714. 30635287 10.1182/blood-2018-11-881268 PMC6473500

[2] Willemze R , Jaffe ES , Burg G , et al. WHO‐EORTC classification for cutaneous lymphomas. Blood. 2005;105(10):3768‐3785. 15692063 10.1182/blood-2004-09-3502

[3] Alaggio R , Amador C , Anagnostopoulos I , et al. The 5th edition of the World Health Organization Classification of Haematolymphoid Tumours: Lymphoid Neoplasms. Leukemia. 2022;36(7):1720‐1748. 35732829 10.1038/s41375-022-01620-2 PMC9214472

[4] Campo E , Jaffe ES , Cook JR , et al. The International Consensus Classification of Mature Lymphoid Neoplasms: a report from the Clinical Advisory Committee. Blood. 2022;140(11):1229‐1253. 35653592 10.1182/blood.2022015851 PMC9479027

[5] Kim YH , Willemze R , Pimpinelli N , et al. TNM classification system for primary cutaneous lymphomas other than mycosis fungoides and Sezary syndrome: a proposal of the International Society for Cutaneous Lymphomas (ISCL) and the Cutaneous Lymphoma Task Force of the European Organization of Research and Treatment of Cancer (EORTC). Blood. 2007;110(2):479‐484. 17339420 10.1182/blood-2006-10-054601

[6] NCCN Clinical Practice Guidelines in Oncology (NCCN Guidelines®) for Guideline Primary Cutaneous Lymphomas Version 1.2024. © National Comprehensive Cancer Network, Inc. 2023. All rights reserved. To view the most recent and complete version of the guideline, go online to NCCN.org. [Last accessed December 25, 2023].

[7] Cankaya R , Schulz S , Moritz R , et al. Skin directed therapy superior to systemic treatment in primary cutaneous B‐cell lymphoma? A study from the Charité; cutaneous lymphoma registry on time to next treatment. Eur J Cancer. 2022;173:S51.

[8] Hamilton SN , Wai ES , Tan K , et al. Treatment and outcomes in patients with primary cutaneous B‐cell lymphoma: the BC Cancer Agency experience. Int J Radiat Oncol Biol Phys. 2013;87(4):719‐725. 24001373 10.1016/j.ijrobp.2013.07.019

[9] Senff NJ , Hoefnagel JJ , Jansen PM , et al. Reclassification of 300 primary cutaneous B‐Cell lymphomas according to the new WHO‐EORTC classification for cutaneous lymphomas: comparison with previous classifications and identification of prognostic markers. J Clin Oncol. 2007;25(12):1581‐1587. 17353548 10.1200/JCO.2006.09.6396

[10] Senff NJ , Hoefnagel JJ , Neelis KJ , et al. Results of radiotherapy in 153 primary cutaneous B‐Cell lymphomas classified according to the WHO‐EORTC classification. Arch Dermatol. 2007;143(12):1520‐1526. 18087001 10.1001/archderm.143.12.1520

[11] Zinzani PL , Quaglino P , Pimpinelli N , et al. Prognostic factors in primary cutaneous B‐cell lymphoma: the Italian Study Group for Cutaneous Lymphomas. J Clin Oncol. 2006;24(9):1376‐1382. 16492713 10.1200/JCO.2005.03.6285

[12] Grange F , Joly P , Barbe C , et al. Improvement of survival in patients with primary cutaneous diffuse large B‐cell lymphoma, leg type, in France. JAMA Dermatol. 2014;150(5):535‐541. 24647650 10.1001/jamadermatol.2013.7452

[13] Porkert S , Mai P , Jonak C , et al. Long‐term Therapeutic Success of Intravenous Rituximab in 26 Patients with Indolent Primary Cutaneous B‐cell Lymphoma. Acta Derm Venereol. 2021;101(2):adv00383. 33475146 10.2340/00015555-3746 PMC9366677

[14] Valencak J , Weihsengruber F , Rappersberger K , et al. Rituximab monotherapy for primary cutaneous B‐cell lymphoma: response and follow‐up in 16 patients. Ann Oncol. 2009;20(2):326‐330. 18836086 10.1093/annonc/mdn636

[15] Campbell BA , Scarisbrick JJ , Kim YH , et al. Time to Next Treatment as a Meaningful Endpoint for Trials of Primary Cutaneous Lymphoma. Cancers (Basel). 2020;12(8):2311. 32824427 10.3390/cancers12082311 PMC7463470

[16] Grange F , Bekkenk MW , Wechsler J , et al. Prognostic factors in primary cutaneous large B‐cell lymphomas: a European multicenter study. J Clin Oncol. 2001;19(16):3602‐3610. 11504742 10.1200/JCO.2001.19.16.3602

[17] Smith BD , Smith GL , Cooper DL , Wilson LD . The cutaneous B‐cell lymphoma prognostic index: a novel prognostic index derived from a population‐based registry. J Clin Oncol. 2005;23(15):3390‐3395. 15908651 10.1200/JCO.2005.08.137

[18] Grange F , Beylot‐Barry M , Courville P , et al. Primary cutaneous diffuse large B‐cell lymphoma, leg type: clinicopathologic features and prognostic analysis in 60 cases. Arch Dermatol. 2007;143(9):1144‐1150. 17875875 10.1001/archderm.143.9.1144

[19] Mian M , Marcheselli L , Luminari S , et al. CLIPI: a new prognostic index for indolent cutaneous B cell lymphoma proposed by the International Extranodal Lymphoma Study Group (IELSG 11). Ann Hematol. 2011;90(4):401‐408. 20872000 10.1007/s00277-010-1083-1

[20] Grange F , Petrella T , Beylot‐Barry M , et al. Bcl‐2 protein expression is the strongest independent prognostic factor of survival in primary cutaneous large B‐cell lymphomas. Blood. 2004;103(10):3662‐3668. 14726400 10.1182/blood-2003-08-2726

[21] Dobos G , de Masson A , Ram‐Wolff C , et al. Epidemiological changes in cutaneous lymphomas: an analysis of 8593 patients from the French Cutaneous Lymphoma Registry. Br J Dermatol. 2021;184(6):1059‐1067. 33131055 10.1111/bjd.19644

[22] Oken MM , Creech RH , Tormey DC , et al. Toxicity and response criteria of the Eastern Cooperative Oncology Group. Am J Clin Oncol. 1982;5(6):649‐655. 7165009

[23] Field A . Discovering Statistics Using SPSS. 3rd ed.: London, California, New Delhi and Singapore, Sage Publications; 2009.

[24] Lucioni M , Berti E , Arcaini L , et al. Primary cutaneous B‐cell lymphoma other than marginal zone: clinicopathologic analysis of 161 cases: Comparison with current classification and definition of prognostic markers. Cancer Med. 2016;5(10):2740‐2755. 27665744 10.1002/cam4.865 PMC5083727

[25] Martínez‐Banaclocha N , Martínez‐Madueño F , Caballé B , et al. A Descriptive Study of 103 Primary Cutaneous B‐Cell Lymphomas: Clinical and Pathological Characteristics and Treatment from the Spanish Lymphoma Oncology Group (GOTEL). Cancers (Basel). 2024;16(5):1034. 38473391 10.3390/cancers16051034 PMC10931196

[26] Hallermann C , Niermann C , Fischer RJ , Schulze HJ . New prognostic relevant factors in primary cutaneous diffuse large B‐cell lymphomas. J Am Acad Dermatol. 2007;56(4):588‐597. 17289214 10.1016/j.jaad.2006.12.026

[27] Hallermann C , Niermann C , Fischer RJ , Schulze HJ . Survival data for 299 patients with primary cutaneous lymphomas: a monocentre study. Acta Derm Venereol. 2011;91(5):521‐525. 21547335 10.2340/00015555-1112

[28] Golling P , Cozzio A , Dummer R , et al. Primary cutaneous B‐cell lymphomas – clinicopathological, prognostic and therapeutic characterisation of 54 cases according to the WHO‐EORTC classification and the ISCL/EORTC TNM classification system for primary cutaneous lymphomas other than mycosis fungoides and Sezary syndrome. Leuk Lymphoma. 2008;49(6):1094‐1103. 18569636 10.1080/10428190802064925

[29] Kim YH , Bagot M , Pinter‐Brown L , et al. Mogamulizumab versus vorinostat in previously treated cutaneous T‐cell lymphoma (MAVORIC): an international, open‐label, randomised, controlled phase 3 trial. Lancet Oncol. 2018;19(9):1192‐1204. 30100375 10.1016/S1470-2045(18)30379-6

[30] Rice MS , Naeger S , Singh E . Real‐World Treatment Patterns and Outcomes Among Multiple Myeloma Patients with Asthma and COPD in the United States. Oncol Ther. 2021;9(1):195‐212. 33728584 10.1007/s40487-021-00146-4 PMC8140164

[31] Perry A , Vincent BJ , Parker SR . Intralesional corticosteroid therapy for primary cutaneous B‐cell lymphoma. Br J Dermatol. 2010;163(1):223‐225. 20394622 10.1111/j.1365-2133.2010.09798.x

[32] Dippel E , Assaf C , Becker JC , et al. S2k‐Leitlinie ‐ Kutane Lymphome (ICD10 C82‐C86): Update 2021. J Dtsch Dermatol Ges. 2022;20(4):537‐555. 10.1111/ddg.14706_g 35446484

[33] Scarisbrick JJ , Quaglino P , Prince HM , et al. The PROCLIPI international registry of early‐stage mycosis fungoides identifies substantial diagnostic delay in most patients. Br J Dermatol. 2019;181(2):350‐357. 30267549 10.1111/bjd.17258

[34] Leiter U , Weichenthal M . [ADOReg –registry of the Scientific Work Group on Dermatological Oncology]. J Dtsch Dermatol Ges. 2014;12(12):1156‐1157. 25482714 10.1111/ddg.12556

