# Supplementary material for: Therapiestrategien bei primär kutanen B‐Zell‐Lymphomen – Ergebnisse einer monozentrischen Kohortenstudie an 98 Patienten
Source: J Dtsch Dermatol Ges. 2025 Jul 14;23(7):822–31. [Article in German] doi: 10.1111/ddg.15702_g (PMC12257065; doi:10.1111/ddg.15702_g)
Supplement: Supplementary file 1 — Supplementary information [file DDG-23-822-s001.docx]

**Online-Abbildungen**

**Online-Abbildung 1.** Kaplan-Meier-Kurve zur Beinbeteiligung bei primär kutanem B-Zell-Lymphom (univariate Analyse)

**Online-Abbildung 2.** Kaplan-Meier-Kurve zur Armbeteiligung bei primär kutanem B-Zell-Lymphom (univariate Analyse)


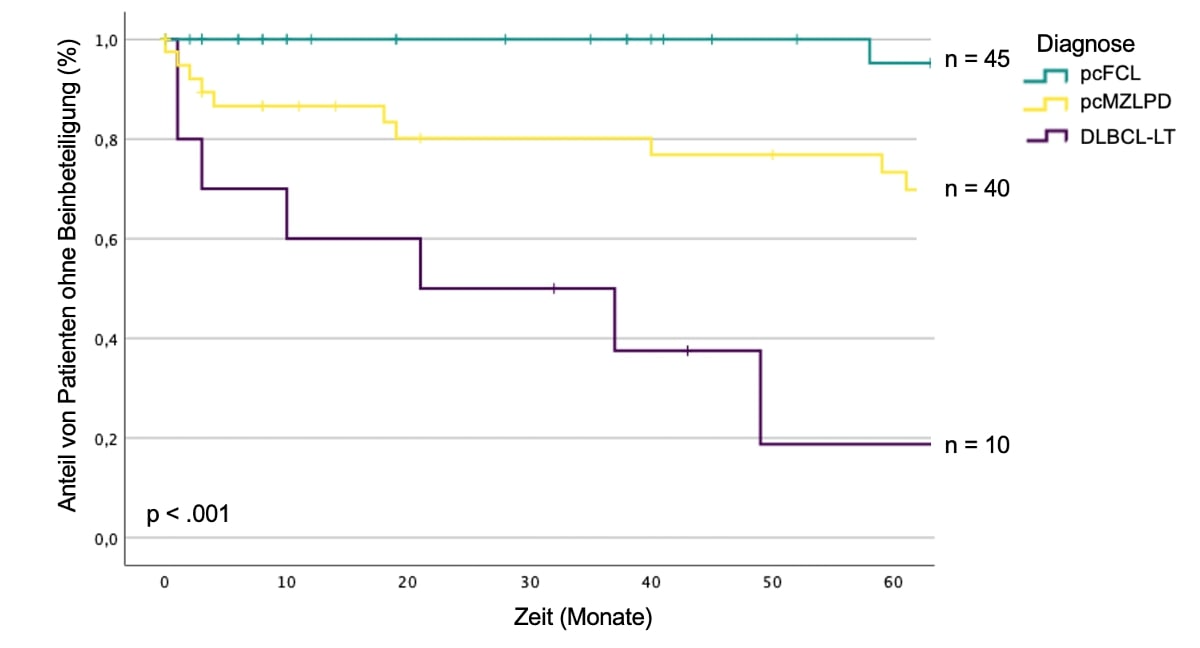
**Online-Abbildung 1.** Kaplan-Meier-Kurve zur Beinbeteiligung bei primär kutanem B-Zell-Lymphom (univariate Analyse)

***Abkürzungen:***

pcFCL, Primär kutanes Follikelzentrum-Lymphom.

pcMZLPD, Primär kutane marginalzonen-lymphoproliferative Störung.

DLBCL-LT, Diffus großzelliges B-Zell-Lymphom, Bein-Typ.

p, P-Wert (Signifikanzniveau: α = 0,05).

n, Anzahl der Patienten.

**Online-Abbildung 2.** Kaplan-Meier-Kurve zur Armbeteiligung bei primär kutanem B-Zell-Lymphom (univariate Analyse)***
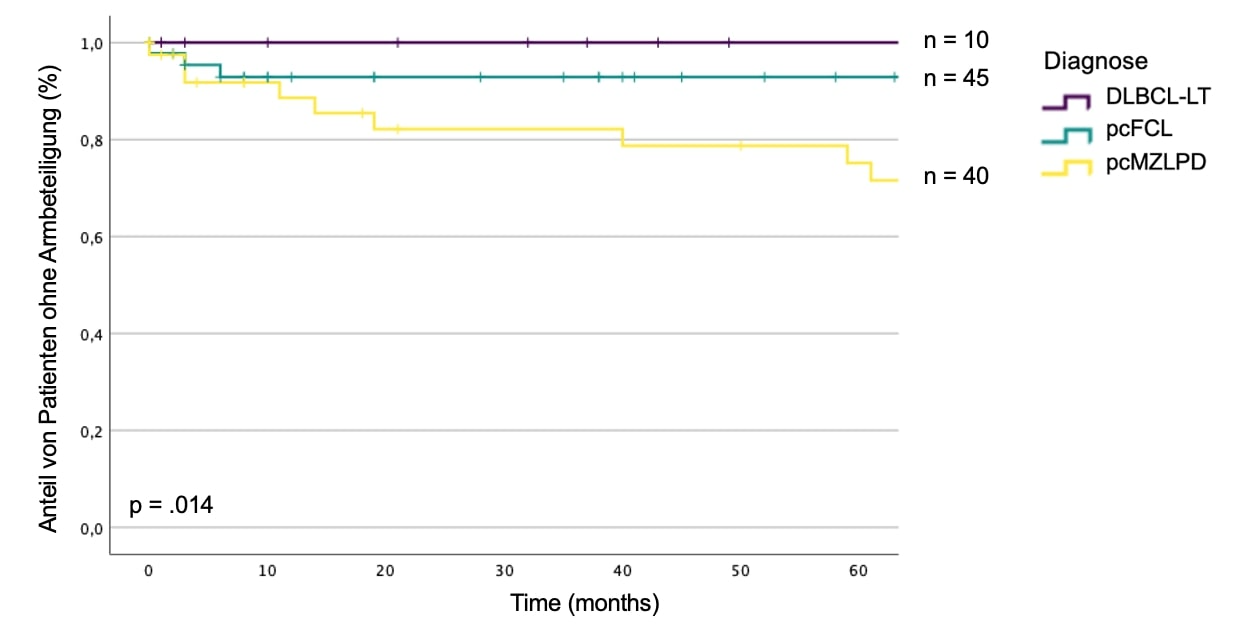
***

**Abkürzungen:**

pcFCL, Primär kutanes Follikelzentrum-Lymphom.

pcMZLPD, Primär kutane marginalzonen-lymphoproliferative Störung.

DLBCL-LT, Diffus großzelliges B-Zell-Lymphom, Bein-Typ.

p, P-Wert (Signifikanzniveau: α = 0,05).

n, Anzahl der Patienten.
